# Reply to van den Bergh and Savin: Fossil fuel taxes are politically hard to change

**DOI:** 10.1073/pnas.2302318120

**Published:** 2023-03-27

**Authors:** Cesar B. Martinez-Alvarez, Chad Hazlett, Paasha Mahdavi, Michael L. Ross

**Affiliations:** ^a^Department of Political Science, University of California, Los Angeles, CA 90095; ^b^Department of Statistics, University of California, Los Angeles, CA 90095; ^c^Department of Political Science, University of California, Santa Barbara, CA 93106

Van den Bergh and Savin (1) offer two criticisms of our study (2): that it “rests on feeble grounds” empirically and that a policy we recommend—scaling-up support for renewable energy—is less effective than carbon pricing. Both are misplaced.

Our study investigates the role of political leadership in raising taxes on, and reducing subsidies for, gasoline. We find that the impact of leaders appears to be small and ephemeral and that most reforms dissipate within a year. This implies that leaders find it extraordinarily hard to meaningfully raise fossil fuel taxes or reduce subsidies.

We would have included post-2015 data if they were available, but they were not. There is no evidence to support the statement that carbon pricing became more effective after 2015; in fact, many countries reduced carbon taxes in 2022 when global prices rose. (3) We employed ordinary least squares with fixed effects (OLS-FE) in our main analysis, where the model’s job was simply to use leader tenure timings to predict net taxes. We made no inferences about coefficients in this model: We simply compared the variance explained by the “true” model to the variance explained by the models with “scrambled” leader timings. Matters of homoscedasticity, normality, or outliers are irrelevant.

We focus on gas taxes and subsidies because they are directly observable and used by all governments and are a natural target for policy recommendations. Other forms of carbon pricing like the EU emissions-trading system (EU-ETS) are used by a much smaller, self-selected group of countries, and so, less of general interest can be said from their experiences. Moreover, a 2022 study finds that carbon pricing data from the EU-ETS are of such low quality that they are “not a good basis for international comparison of policy effectiveness (4).”

Van den Bergh and Savin imply that we favor a “renewables only” policy as an alternative to carbon pricing, but we do not. We would argue—as many others do—that no single policy will bring about the rapid decarbonization needed to achieve the Paris goals. Our study hence recommends a wide range of climate policies including standards to limit fossil fuel use, investments in public transit, and green industrial policies.

We disagree with some of their criticisms of renewable energy. All types of energy production have net positive life-cycle greenhouse gas emissions, but emissions from solar, wind, and nuclear technologies are considerably lower than emissions from fossil fuel-based technologies (5).

We agree with other points: The historical pace of renewables diffusion is too slow and should be dramatically expanded, and some gains from renewables will be offset by a rebound effect. Their idea for a China–EU–US climate club with a joint carbon price is appealing, but they offer no reason to believe that this is “the most realistic scenario for solving climate change.”

Climate policies cannot be “effective” or “realistic” if they are not politically sustainable. A single-minded focus on carbon pricing—an unpopular and often unsuccessful policy—is a very risky bet.
